# MicroRNA-532-5p is implicated in the regulation of osteoporosis by forkhead box O1 and osteoblast differentiation

**DOI:** 10.1186/s12891-020-03317-y

**Published:** 2020-05-13

**Authors:** Xinyu Guo, Shijun Wei, Feng Xu, Xianhua Cai, Huasong Wang, Ran Ding

**Affiliations:** 1 Department of Orthopaedics, Guizhou Provincial Orthopaedic Hospital, Guiyang City, Guizhou Province 550002 PR China; 2 Orthopedic surgery of Wuhan General Hospital of People’s Liberation Army, No.627 Wuluo Road, Wuhan City, Hubei Province 430000 PR China

**Keywords:** miR-532-5p, FOXO1, Osteoporosis, Osteogenic differentiation

## Abstract

**Background:**

MicroRNAs (miRNAs) are critical regulators in osteogenesis and cartilage formation. This study was designed to investigate whether miR-532-5p plays a role in the regulation of osteoporosis.

**Methods:**

Osteoporotic fractures (OP group, *n* = 10) or osteoarthritis without osteoporosis (control group, *n* = 10) were selected as subjects in this study. Quantitative analysis of gene expression was performed by RT-PCR. Western blot was used to determine the expression levels of protein forkhead O1 (FOXO1). Bioinformatics analyses and luciferase reporter assay were used to verify the downstream target of miR-532-5p.

**Results:**

Compared with the non-osteoporotic controls, miR-532-5p was upregulated in osteoporotic samples, and expression of miR-532-5p was downregulated in the osteogenic C2C12 cell model. Overexpression of miR-532-5p resulted in decreased expression levels of key osteoblast markers, including alkaline phosphatase (ALP), osteocalcin (OC), and collagen type I alpha 1 (COL1A1). The inhibitory results of miR-532-5p were reversed. MiR-532-5p contained a putative FOXO1 binding site. Moreover, miR-532-5p inhibited the expression of FOXO1, and overexpression of FOXO1 inhibited the effect of miR-532-5p on osteoblast markers.

**Conclusions:**

MiR-532-5p can provide references to osteoporosis by regulating the expression of FOXO1 and osteoblast differentiation. MiR-532-5p might serve as a therapeutic target for osteoporosis.

## Background

With the trends in population aging, the incidence of age-related diseases has increased, which requires more attention and resources to manage diseases associated with the elderly. Osteoporosis can lead to bone fragility [1]. One serious consequence of osteoporosis is the occurrence of osteoporotic fracture [2]. The pain and dysfunction caused by fracture significantly affect the quality of life of patients [3]. The prevalence rate of osteoporosis has increased significantly in recent years also due to the increased aging of the global population [4]. Therefore, it is of great social and economic value to study the pathological process of osteoporosis. Osteoporosis is mainly caused by insufficient differentiation of undifferentiated stem cells in the fascia scaffold into osteoblasts after bone tissue absorption [5].

Recent studies have found that miRNAs play critical roles in various pathological processes in the body, including cell apoptosis, biological growth, virus defense, hematopoietic processes, glycolipid metabolism, and disease development [6, 7]. They regulate the proliferation of osteoporosis and gene expression in bone tissue development, and ultimately affect the formation and metabolism of bones [8]. They are also critical regulators of signaling pathways involved in bone development and osteoblast proliferation in bone tissues [9]. MiR-532-5p is located on Xp11.23 of the human chromosome [10]. It was reported to have cancer-promoting effect in cutaneous melanoma [11]. Studies have found that it is down-regulated and can inhibit cell proliferation in a variety of solid diseases [12]. For example, the expression levels of miR-532-5p in rat osteoblasts were decreased after PTH treatment. MiR-532-5p plays a critical role in controlling bone remodeling by MMP-13 [13]. However, the role of miR-532-5p in osteoporosis is still unclear.

MiRNAs have been found to play their biological roles by regulating expression of target genes [14]. Forkhead box protein O (FOXO) is a broad-ranging transcription factor [15]. FOXO1 belongs to the FOX protein family. It regulates various pathophysiological processes such as cell differentiation, DNA damage repair, tumor metabolism, proliferation, and signal transduction [16]. Recent studies have found that the regulation of FOXO1 in bone varies with different cell types [17]. In osteoblasts, FOXO1 protein promotes protein synthesis by interacting with ATF4 to counteract oxidative stress in bone, maintaining normal proliferation of osteoblasts [18]. FOXO1 promotes the differentiation of osteoblast precursors into osteoblasts and may have an inhibitory effect on osteoclasts [18]. Studies have also found that overexpression of FOXO1 reduces the number of osteoblasts [19]. Collagen type I (COL1A1) is the main component of bone matrix and has the function of resisting deformation [20]. Alkaline phosphatase (ALP) is mainly expressed in hypertrophic chondrocytes and osteoblasts during intrachondral osteogenesis [21]. Osteocalcin (OC) is the most abundant collagen in bone. The content of osteocalcin can effectively reflect the activity of osteoblasts [22]. In the present study, the functions of miR-532-5p in the regulation of osteoporosis were studied and its interactions with FOXO1 were also investigated. This study will provide an experimental basis for the search for new drug targets.

## Methods

### Clinical samples

Ten postmenopausal women diagnosed with osteoarthritis without osteoporosis were enrolled in the control group (Supplementary Table 1). Ten postmenopausal women who underwent hip replacement due to osteoporotic fractures (op) were enrolled in the experimental group. Samples of these participants were collected at the Wuhan General Hospital of People’s Liberation Army from March 2018 to February 2019. None of the participants had a history of other disease including metabolic or endocrine disease, chronic renal failure, chronic liver disease, malignancies, Paget’s disease of bone, malabsorption syndrome, hormone replacement therapy, anti-resorptive or anabolic agents, oral corticosteroids, anti-epileptic drugs, or treatment with lithium, heparin, or warfarin. This study was approved by the ethics committee of Wuhan General Hospital of People’s Liberation Army. All participants signed the written informed consent.

Fragments of trabecular bone were obtained from osteoarthritic patients undergoing replacement knee surgery. The patients had no clinical symptoms of bone metabolic disorders. These bone samples were minced into 0.5–1.0 cm^2^ pieces and were washed extensively in phosphate-buffered saline (PBS) to remove adherent bone marrow cells. The bone samples were homogenized in Trizol (Life Technologies) and total RNAs were extracted using the RNeasy kit (Lianshuo, Zhejiang, China). The concentrations of purified RNAs were measured using a spectrophotometer (Jinghua, Shanghai, China).

### Cell culture, differentiation and transfection

C2C12 cells were cultured in DMEM/F12 complete medium containing 10% FBS and were kept in a cell incubator at 37 °C with 5% CO2. Cells were treated with 2 nM BMP-2 (Invitrogen Life Technologies, CA, USA) to induce osteogenic differentiation. MiR-532-5p mimetic/inhibitor, mimetic (NC)/inhibitor control (NC inhibitor) and FOXO1-siRNA (si-FOXO1) (sc-35,382) were obtained from Santa Cruz Biotechnology, Inc. Oligonucleotides and plasmids were transfected with Lipofectamine™ 2000 Transfection Reagent (Invitrogen, USA) for 48 h.

### Quantitative polymerase chain reaction (qPCR)

Total RNAs of cells was extracted using TRIzol reagent (Haigene, Heilongjiang, China). qRT-PCR was performed using a ViiATM 7 real-time PCR system (Jinuo, Shanghai, China). GAPDH and U6 were used as internal references. The expression levels of lncRNA LINC00461 and miR-30a-5p were detected using SYBR Premix Ex Taq II (Takara Biotechnology). qRT-PCR were performed with reference to the literature [23]. Primer sequences were listed in Table 1.

**Table 1:** Sequences of primers used in qRT-PCR

Gene	Forward primer (5′-3′)	Reversed primer (5′-3′)
miR-532-5p	CTTCCATGCCTTGAGTGTA	GTGTGGGAGGGTAATTAAGATG
U6	CTCGCTTCGGCAGCACA	AACGCTTCACGAATTTGCGT
FOXO1	CCAGCCCAAACTACCAAAAATA	GAGGAGAGTCAGAAGTCAGCAAC
OC	CTGACAAAGCCTTCATGTCCAA	GGTAGCGCCGGAGTCTGTT
ALP	GACAAGAAGCCCTTCACTGC	AGACTGCGCCTGGTAGTTGT
COL1A1	GGGTCTAGACATGTTCAGCTTTGTG	ACCCTTAGGCCATTGTGTATGC
GAPDH	ACAACTTTGGTATCGTGGAAGG	GCCATCACGCCACAGTTTC

### ALP staining and measurement

C2C12 cells were harvested, the culture medium was removed and ALP activity was measured using the ALP Colorimetric Assay Kit (Laier, Hefei,China).

### Western blot

Total proteins were extracted and protein concentrations were quantified using the BCA Protein As-say Kit. Protein samples were incubated with rabbit anti-FOXO1 (1:500, Shidai, Shanghai, China) and GAPDH (1500, Shidai, Shanghai, China) for overnight, followed by incubation with anti-rabbit secondary antibody (1,5000) for 1 h. Western blot was performed with reference to the literature [24].

### Luciferase reporter gene assay

Online software starBase (http://starbase.sysu.edu.cn/) was used to identify the target of miR-532-5p. The wild type FOXO1–3′-UTR (WT) and mutant FOXO1–3′-UTR (MT) vectors containing the putative binding site of miRNA-532-5p were constructed. A reporter vector containing WT or MT FOXO13′-UTR was co-transfected into C2C12 cells with miR-345-5p mimic or NC using Lipofectamine™ 2000. After 48 h of transfection, luciferase activity was assessed using a dual-luciferase assay system (Promega).

### Statistical method

Data were analyzed by the SPSS19.0 statistical software. The results of data were shown as mean ± standard deviation (SD). Comparisons among multiple groups were performed based on one-way ANOVA. LSD test was used for subsequent analysis. *P* < 0.05 indicated significant differences.

## Results

### MiR-532-5p was down-regulated during osteogenic differentiation

**Fig. 1:**
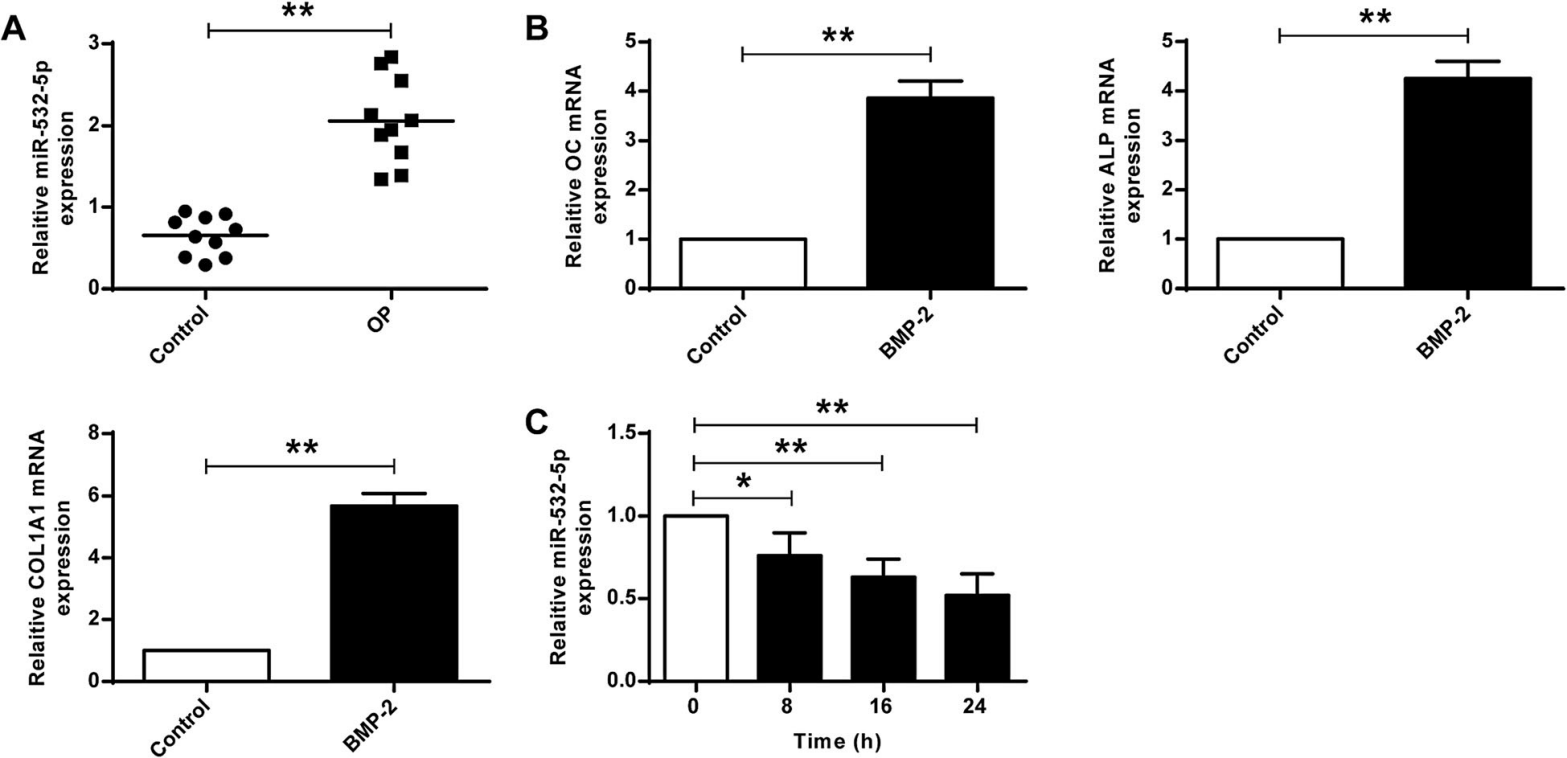
The levels of MiR-532-5p during OP and osteogenic differentiation. **a** The levels of miR-532-5p in osteoporotic fractures and control patients. **b** BMP2-induced levels of OC, ALP and COL1A1 in C2C12 cells. **c** BMP-2 treatment of cells for 0, 8, 16 and 24 h, after which the expression level of miR-532-5p was determined.** *P* < 0.01

As shown in Fig. 1a, compared with the control group, the expression levels of miR-532-5p were significantly increased in OP patients (*P* < 0.01), indicating that miR-532-5p plays a part in the progression of osteoporosis. As shown in Fig. 1b, the expression levels of osteoblast markers (OC, ALP and COL1A1) were significantly higher in BMP2-treated C2C12 cells (*P* < 0.01), indicating successful induction of osteogenesis. As shown in Fig. 1c, in BMP2-treated C2C12 cells, the expression of miR-532-5p was significantly down-regulated (*P* < 0.05, *P* < 0.01). These results demonstrated that miR-532-5p was involved in the differentiation of osteogenic.

### Effects of miR-532-5p on osteoblast differentiation in C2C12 cells

**Fig. 2:**
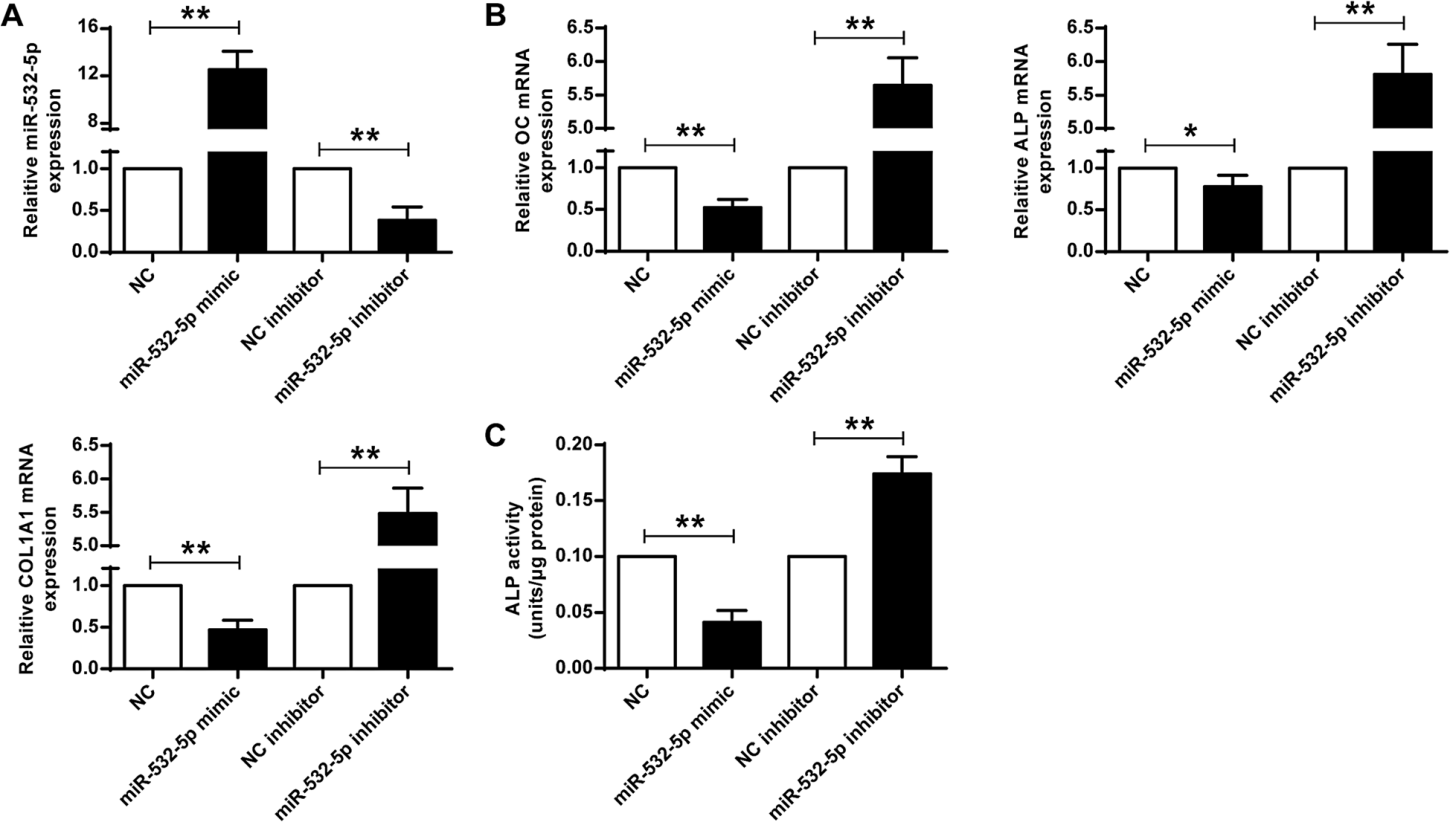
MiR-532-5p inhibited osteogenic differentiation of C2C12 cells. **a** expression levels of miR-532-5p in C2C12 cells. **b** mRNA levels of OC, ALP and COL1A1 in C2C12 cells. **c** ALP activity in C2C12 cells. * *P* < 0.05, ** *P* < 0.01

As shown in Fig. 2a, compared with the control group, the expression levels of miR-532-5p were significantly higher in the miR-532-5p mimic group. The expression levels of miR-532-5p were significantly decreased in the miR-532-5p inhibitor group (*P* < 0.05), indicating successful transfection. And the expression levels of OC, ALP and COL1A1 in the miR-532-5p mimic group were significantly decreased, and the expression levels of OC, ALP and COL1A1 in the miR-532-5p inhibitor group were significantly higher (*P* < 0.05) (Fig. 2b). ALP activity was significantly reduced in the miR-532-5p mimic group, and ALP activity was significantly increased in the miR-532-5p inhibitor group (*P* < 0.05) (Fig. 2c). These results demonstrated that miR-532-5p can inhibit osteogenic differentiation.

### FOXO1 was a target of miR-532-5p

**Fig. 3:**
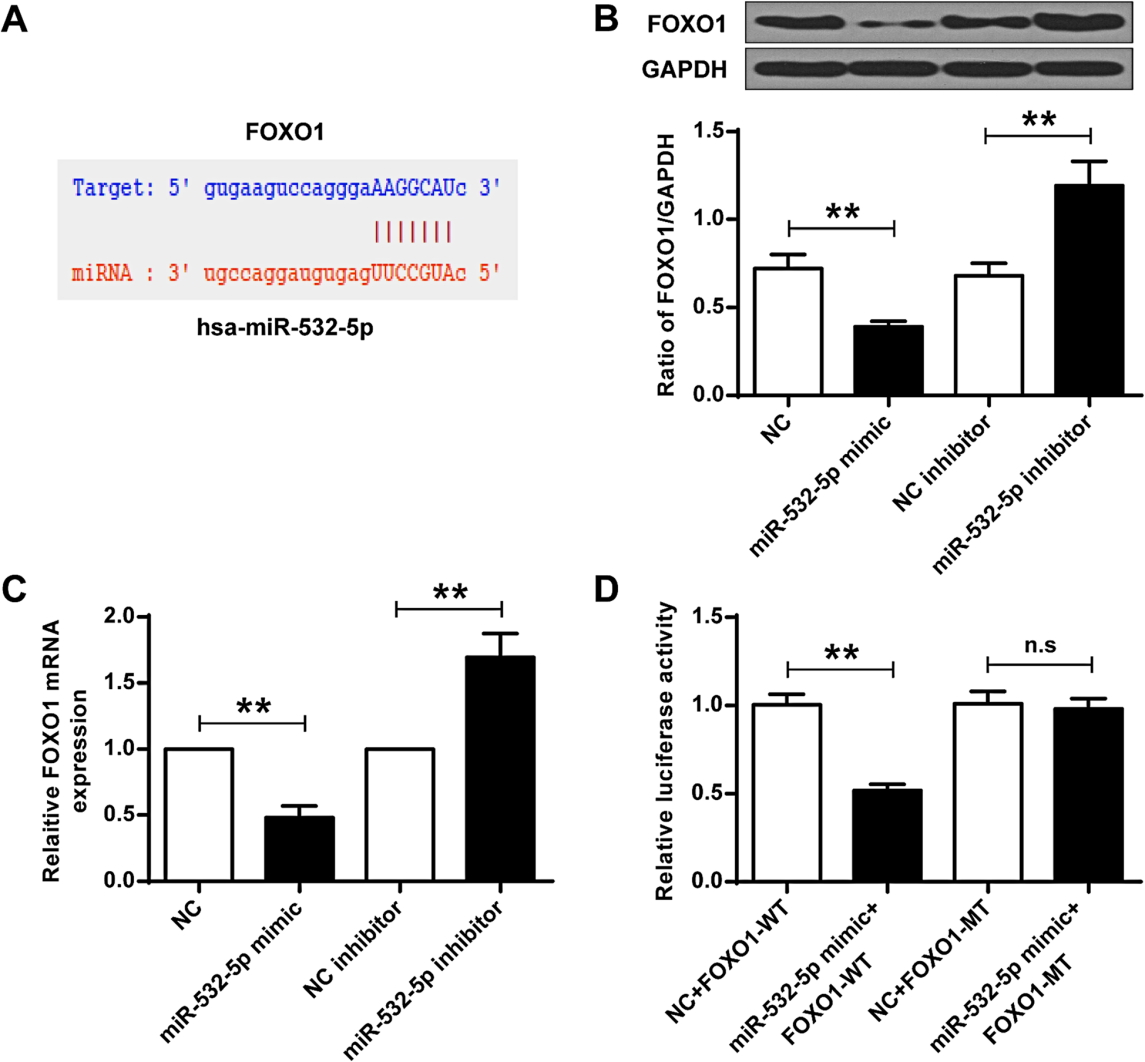
FOXO1 was the target of miR-532-5p. **a** Putative target sequence of FOXO1 on the 3′-UTR of miR-532-5p.(**b**) Protein expression level of FOXO1 in C2C12 cells. **c** Expression levels of FOXO1 in C2C12 cells. **d** Luciferase activity assay after co-transfection of C2C12 cells with a miR-532-5p mimetic and a reporter plasmid containing wild type (WT) or Mut (MT) 3’-UTR of FOXO1. ** *P* < 0.01

To identify the target genes of miR-532-5p in osteogenesis, the miRNA target prediction database starBase (http://starbase.sysu.edu.cn/) was used and FOXO1 was identified as a potential target for miR-532-5p (Fig. 3a). As shown in Fig. 3b and c, the expression levels of FOXO1 protein were significantly decreased in the miR-532-5p mimic group compared to that in the NC group (*P* < 0.01), while the expression levels of FOXO1 protein in the miR-532-5p inhibitor group were significantly increased (*P* < 0.01). To validate these results, C2C12 cells were co-transfected with the miR-532-5p mimic and the wild-type (WT) or Mut (MT) 3′-UTR containing FAXO1 and luciferase activity assay was performed. As shown in Fig. 3d, miR-532-5p significantly inhibited the activity of the FOXO1-WT reporter gene (*P* < 0.01), but the activity of the mutant reporter gene was not affected (*P* > 0.05). These data indicated that miR-532-5p may exert its biological functions through FOXO1.

### MiR-532-5p inhibits osteogenic differentiation through FOXO1

**Fig. 4:**
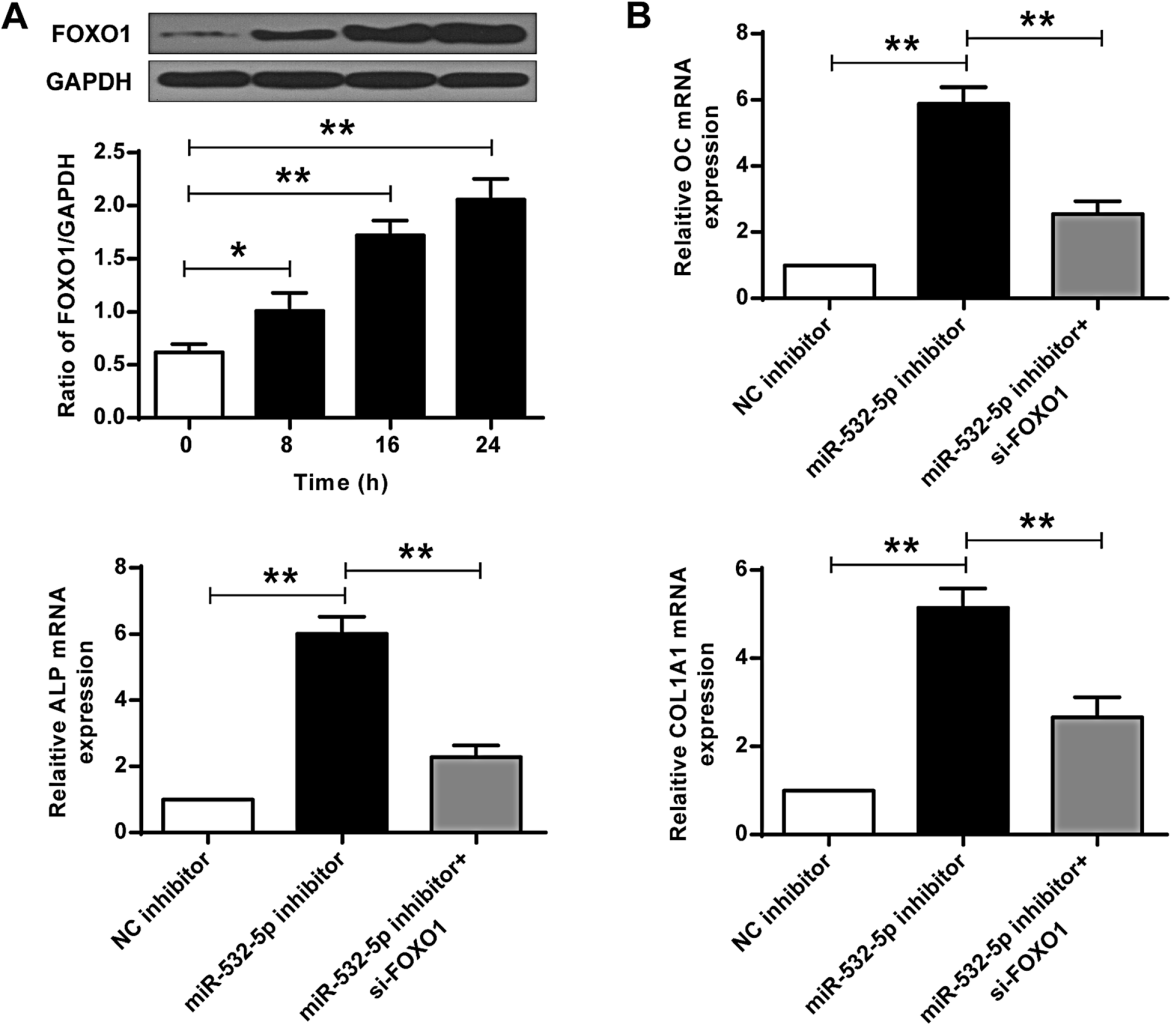
MiR-532-5p inhibited osteogenic differentiation of C2C12 cells by targeting FOXO1. **a** BMP-2 induced FOXO1 protein expression levels in C2C12 cells. **b** Expression levels of OC, ALP and COL1A1 in C2C12 cells. * *P* < 0.05, ** *P* < 0.01

The expression levels of FOXO1 protein in C2C12 cells after BMP-2 stimulation were then examined. The expression of FOXO1 was significantly upregulated in BMP2-treated C2C12 cells (*P* < 0.01) (Fig. 4a). In addition, the expression levels of osteoblast markers (OC, ALP, and COL1A1) were significantly raised in the miR-532-5p inhibitor group compared with that in the NC inhibitor group. Co-transfection with FOXO1 siRNA significantly reduced the expression levels of COL1A1, OC and ALP (*P* < 0.01) (Fig. 4b). These results demonstrated that miR-532-5p inhibited osteogenic differentiation by down-regulating FOXO1.

## Discussion

Osteoporosis (OP) is a systemic chronic metabolic disease, occurring mostly in postmenopausal women and the elderly [25]. In recent years, OP has become a worldwide problem that seriously jeopardizes public health, and its incidence has been ranked the first among middle-aged and elderly orthopedic diseases [26]. The main pathological characteristic of osteoporosis is inadequate bone formation during bone reconstruction [27]. The osteoblastic bone formation is an important core cell for maintaining normal bone metabolism. Therefore, osteoblasts are of great significance in patients with osteoporosis or other pathological osteoporosis.

Studies have shown that miRNAs participate in various important biological processes, including cell proliferation, differentiation, apoptosis and development [28]. It has been confirmed that many miRNAs play critical roles in bone metabolism [29]. The discovery of specific microRNAs and the underlying mechanisms of their regulation in bone metabolism involves important clinical issues, such as fracture treatment, disease of osteoporosis and osteoarthritis [30]. Recent studies found that miRNAs are also involved in the differentiation and development of osteoblasts [31]. For example, the expression of miR-346 was upregulated in the hBMSCs into osteoblasts, and it could bind to glycogen synthetic kinase 313 (GSK313) to regulate the Wnt/β-catenin signaling pathway, which promoted the differentiation of osteoblasts [32]. It was reported that the expression level of miR-532-5p in osteoblasts was significantly reduced after 4 h [13]. Our study found that miR-532-5p was downregulated in OP patients was reduced. These indicated that miR-532-5p was involved in the progression of osteoporosis.

During the differentiation of osteoblast, various osteoblast-specific genes, such as COL1A1, ALP and OC, are expressed at different stages [33]. COL1A1 is secreted by osteoblasts and is the main organic component of bone matrix. It plays a part in biomechanical properties of bone structure. The expression of COL1A1 can be considered as a molecular marker for bone formation and bone remodeling [34]. ALP is a calcium-binding transporter mainly distributed in cell membrane, and its expression gradually increases with the degree of cell differentiation, which is an early indicator of osteoblast differentiation [35]. OC is a non-collagen protein that is specifically synthesized and secreted by osteoblasts. It is an essential factor for bone calcification [36]. This study found that in BMP2-treated C2C12 cells, the expression of miR-532-5p was downregulated. The expression levels of OC, ALP and COL1A1 and the ALP activity in the miR-532-5p mimic group were decreased compared with the control group, and the results in the miR-532-5p inhibitor group were opposite. These results demonstrated that miR-532-5p can inhibit osteogenic differentiation.

Studies have found that miRNAs affect the occurrence and development of diseases through regulation of different signaling pathways. FOXO1 plays important roles in many biological processes such as apoptosis, stress, DNA damage/repair, tumorigenesis, angiogenesis and glucose metabolism, and bone metabolism [37]. In the osteoblast cell line, FOXO1 regulates redox balance, protein synthesis, and osteogenic differentiation of cells through interactions with transcriptional activator 4 (ATF4), bone-specific transcription factor (Runx2) and ALP [38]. The FOXO1 in osteogenic differentiation has become a research hotspot. Recent studies found that protein accumulation of FOXO1 was decreased in BMMSCs of ovariectomized mice, which was caused by accumulation of tumor necrosis factor-alpha (TNF-α) after estrogen deficiency. Mechanistically, TNF-α activated NF-κB pathway to promote the expression of microRNA-705, which functions as a repressor of FOXO1 through post-transcriptional regulation. Inhibition of NF-κB pathway or knockdown of miR-705 largely prevented the decreasing of FOXO1-mediated antioxidant defense caused by TNF-α and ameliorated the oxidative damage in osteoporotic BMMSCs [39]. This study found that expression of FOXO1 protein was significantly up-regulated in BMP2-treated C2C12 cells. FOXO1 may be a downstream target gene of miR-532-5p. In addition, co-transfection with FOXO1 siRNA in the miR-532-5p inhibitor group significantly reduced the expression levels of OC, ALP and COL1A1. These results demonstrated that miR-532-5p was able to suppress osteogenic differentiation by down-regulating the expression of FOXO1. Further investigations are needed to prove the value of miR-532-5p as a biomarker and a therapeutic target osteoporosis.

## Conclusion

MiR-532-5p had a critical function in osteoporosis by regulating FOXO1 and osteoblast differentiation. It suggests that miR-532-5p can be a potential therapeutic target for osteoporosis. It would provide experimental evidence for the clinical prognosis of the disease and targeted intervention therapy.

## Supplementary information

**Additional file 1.**

## Data Availability

The datasets used and/or analyzed during the current study are available from the corresponding author on reasonable request.

## Abbreviations

miRNAs: MicroRNAs
ALP: Alkaline phosphatase
OC: Osteocalcin
COL1A1: Collagen type I alpha 1
FOXO1: Forkhead O1
qPCR: Quantitative polymerase chain reaction
FOXO: Forkhead box protein O
si-FOXO1: FOXO1-siRNA
OP: Osteoporosis
GSK313: Glycogen synthetic kinase 313
ATF4: Transcriptional activator 4
Runx2: Bone-specific transcription factor
GSK313: Glycogen synthetic kinase 313
TNF-α: Tumor necrosis factor-alpha

## References

[1] Chen X, Su JC (2017). New focus on osteoporosis: differentiation fate of bone marrow-derived mesenchymal stem cells. Acad J Second Mil Univ.

[2] Gielen E, Bergmann P, Bruyere O, Cavalier E, Delanaye P, Goemaere S (2017). Osteoporosis in frail patients: a consensus paper of the Belgian bone Club. Calcif Tissue Int.

[3] Rizzoli R (2011). Bisphosphonates for post-menopausal osteoporosis: are they all the same?. QJM..

[4] Varahra A, Rodrigues IB, MacDermid JC, Bryant D, Birmingham T (2018). Exercise to improve functional outcomes in persons with osteoporosis: a systematic review and meta-analysis. Osteoporos Int.

[5] Khosla S, Cauley JA, Compston J, Kiel DP, Rosen C, Saag KG (2017). Addressing the crisis in the treatment of osteoporosis: a path forward. J Bone Miner Res.

[6] Tomankova T, Petrek M, Kriegova E (2010). Involvement of microRNAs in physiological and pathological processes in the lung. Respir Res.

[7] Yang F, Cheng Y, Cao Y, Dong H, Lu H, Zhang K (2019). Sensitively distinguishing intracellular precursor and mature microRNA abundance. Chem Sci.

[8] Zeng HC, Bae Y, Dawson BC, Chen Y, Bertin T, Munivez E (2017). MicroRNA miR-23a cluster promotes osteocyte differentiation by regulating TGF-beta signalling in osteoblasts. Nat Commun.

[9] Zhu XB, Lin WJ, Lv C, Wang L, Huang ZX, Yang SW (2018). MicroRNA-539 promotes osteoblast proliferation and differentiation and osteoclast apoptosis through the AXNA-dependent Wnt signaling pathway in osteoporotic rats. J Cell Biochem.

[10] Song X, Wang Z, Jin Y, Wang Y, Duan W (2015). Loss of miR-532-5p in vitro promotes cell proliferation and metastasis by influencing CXCL2 expression in HCC. Am J Transl Res.

[11] Kitago M, Martinez SR, Nakamura T, Sim MS, Hoon DS (2009). Regulation of RUNX3 tumor suppressor gene expression in cutaneous melanoma. Clin Cancer Res.

[12] Griesing S, Kajino T, Mei CT, Liu Z, Takahashi T. TTF-1-regulated miR-532-5p targets KRAS and MKL2 oncogenes and induces apoptosis in lung adenocarcinoma cells. Cancer Sci. 2017;108(7). 10.1111/cas.13271 PMC5497805 28474808

[13] Vishal, Mohanakrishnan, Arumugam, Balasubramanian, Gokulnath, Mahalingam, et al. Parathyroid hormone-induced down-regulation of miR-532-5p for matrix metalloproteinase-13 expression in rat osteoblasts. 10.1002/jcb.26827 PMC7461727 29626351

[14] Lotfi A, Pervaiz T, Jiu S, Faghihi F, Jahanbakhshian Z, Khorzoghi EG (2017). Role of microRNAs and their target genes in salinity response in plant s.

[15] Link W, Fernandez-Marcos PJ. FOXO transcription factors at the interface of metabolism and cancer. Int J Cancer. 2017. 10.1002/ijc.30840 28631330

[16] Jiang S, Li T, Yang Z, Hu W, Yang Y. Deciphering the roles of FOXO1 in human neoplasms. Int J Cancer. 2018. 10.1002/ijc.31338 29473160

[17] Jiang Z, Tian J, Zhang W, Yan H, Liu L, Huang Z (2017). Forkhead protein FoxO1 acts as a repressor to inhibit cell differentiation in human fetal pancreatic progenitor cells. J Diabetes Res.

[18] Dixit M, Singh KB, Prakash R, Singh D (2017). Functional block of IL-17 cytokine promotes bone healing by augmenting FOXO1 and ATF4 activity in cortical bone defect model. Osteoporos Int.

[19] Lou Z, Peng Z, Wang B, Li X, Li X, Zhang X (2019). miR-142-5p promotes the osteoclast differentiation of bone marrow-derived macrophages via PTEN/PI3K/AKT/FoxO1 pathway. J Bone Miner Metab.

[20] Rujitanaroj PO, Jao B, Yang J, Wang F, Anderson JM, Wang J, et al. Controlling fibrous capsule formation through long-term down-regulation of collagen type I (COL1A1) expression by nanofiber-mediated siRNA gene silencing. Acta Biomater. 9(1):4513–24. 10.1016/j.actbio.2012.09.029 PMC3523808 23036951

[21] Mornet E, Fo M, Ngo S, Taillandier A, Simon-Bouy B, Maire I, et al. Correlation of alkaline phosphatase (ALP) determination and analysis of the tissue non-specific ALP gene in prenatal diagnosis of severe hypophosphatasia. Prenat Diagn. 19(8):755–7. 10.1002/(sici)1097-0223(199908)19:8<755::aid-pd629>3.0.co;2-# 10451522

[22] Wada S, Kamiya S (2006). Bone and bone related biochemical examinations. Bone and collagen related metabolites. Osteocalcin (OC). Clinical Calcium.

[23] Hürkan K, Sezer F, Özbilen A, Taşkın KM (2018). Biotechnology. Identification of reference genes for real-time quantitative polymerase chain reaction based gene expression studies on various olive (Olea europaea L.) tissues. J Horticultural Sci.

[24] Swets M, Wouters A, Krijgsman D, van Vlierberghe RLP, Boot A, van Eendenburg JD (2018). HLA-G protein expression in colorectal cancer evaluated by immunohistochemistry and western blot analysis: its expression characteristics remain enigmatic. Clin Immunol.

[25] Kuo TR, Chen CH (2017). Bone biomarker for the clinical assessment of osteoporosis: recent developments and future perspectives. Biomark Res.

[26] Adebusoye LA, Ogunbode AM, Olowookere OO, Ladipo M, Balogun WO, Alonge TO (2017). Factors associated with osteoporosis among older patients at the geriatric Centre in Nigeria: a cross-sectional study. Official J S Afr Acad Fami Pract Prim Care.

[27] Kathuria P, Gordon KB, Silverberg JI. Association of psoriasis and psoriatic arthritis with osteoporosis and pathological fractures. Retour Au Numéro. 2017;76(6). 10.1016/j.jaad.2016.11.046 28314685

[28] Vidal DO, Ramao A, Pinheiro DG, Muys BR, Lorenzi JCC, de Padua AC (2018). Highly expressed placental miRNAs control key biological processes in human cancer cell lines. Oncotarget..

[29] Yuan Y, Zhang L, Tong X, Zhang M, Zhao Y, Guo J (2017). Mechanical stress regulates bone metabolism through MicroRNAs. J Cell Physiol.

[30] Taipaleenmäki H (2018). Regulation of bone metabolism by microRNAs. Current Osteoporosis Reports.

[31] Seenprachawong K, Nuchnoi P, Nantasenamat C, Prachayasittikul V, Supokawej A (2016). Computational identification of miRNAs that modulate the differentiation of mesenchymal stem cells to osteoblasts.

[32] Wang Q, Cai J, Cai XH, Chen L (2013). miR-346 regulates osteogenic differentiation of human bone marrow-derived mesenchymal stem cells by targeting the Wnt/beta-catenin pathway. PLoS One.

[33] Zhang X, Liu YS, Lv LW, Chen T, Wu G, Zhou YS (2016). Promoted role of bone morphogenetic protein 2/7 heterodimer in the osteogenic differentiation of human adipose-derived stem cells. Beijing Da Xue Xue Bao.

[34] Soibam D, Singh TA, Nandy P, Dewan SK, Baruah A (2018). Sp1 binding site polymorphism at COL1A1 gene and its relation to bone mineral density for osteoporosis risk factor among the Sikkimese men and women of Northeast India. Indian J Clin Biochem.

[35] Huang CX, Yu HL, Gao C, Liu YD. Effect of low-level laser irradiation (LLLI) combined with ferulic acid on the osteoblast differentiation and maturation as well as osteogenesis signaling pathway expression. J Hainan Med Univ. 2017;23(18).

[36] Zhang RF, Wang Q, Zhang AA, Xu JG, Zhai LD, Yang XM (2018). Low-level laser irradiation promotes the differentiation of bone marrow stromal cells into osteoblasts through the APN/Wnt/beta-catenin pathway. Eur Rev Med Pharmacol Sci.

[37] Hao W, Liu H, Zhou L, Sun Y, Wang X (2017). MiR-145 regulates osteogenic differentiation of human adipose-derived mesenchymal stem cells through targeting FoxO1. Exp Biol Med.

[38] Zhang H, Pan Y, Zheng L, Choe C, Huang H (2011). FOXO1 inhibits Runx2 transcriptional activity and prostate Cancer cell migration and invasion. Cancer Res.

[39] Liao L, Su X, Yang X, Hu C, Li B, Lv Y (2016). TNF-alpha inhibits FoxO1 by Upregulating miR-705 to aggravate oxidative damage in bone marrow-derived Mesenchymal stem cells during osteoporosis. Stem Cells.

