# Supplementary material for: MicroRNA-532-5p is implicated in the regulation of osteoporosis by forkhead box O1 and osteoblast differentiation
Source: BMC Musculoskelet Disord. 2020 May 13;21:296. doi: 10.1186/s12891-020-03317-y (PMC7218624; doi:10.1186/s12891-020-03317-y)
Supplement: Supplementary file 1 — Additional file 1. [file 12891_2020_3317_MOESM1_ESM.doc]

**Supplementary Table 1**

Patient characteristics

|  | n | Age (years) | T-score | BMD (g/cm^2^) |
| --- | --- | --- | --- | --- |
| Osteoporotic | 10 | 62.15±3.54 | -3.48±0.31 | 0.49±0.08 |
| Non-osteoporotic | 10 | 63.44±6.07 | 0.49±0.51 | 0.83±0.05 |
